# Tracking Intracellular Labile Iron with a Genetically Encoded Fluorescent Reporter System Based on Protein Stability

**DOI:** 10.1021/acssensors.5c01165

**Published:** 2025-08-01

**Authors:** Ali Akyol, Şeyma Çimen, Benjamin Gottschalk, Yusuf C. Erdoğan, Anna Lischnig, Amy Barton Alston, Reinaldo Digigow, Beat Flühmann, Emrah Eroğlu, Wolfgang F. Graier, Roland Malli

**Affiliations:** † Gottfried Schatz Research Center, Molecular Biology and Biochemistry, 31475 Medical University of Graz, Neue Stiftingtalstraße 6, 8010 Graz, Austria; ‡ Regenerative and Restorative Medicine Research Center (REMER), Research Institute for Health Sciences and Technologies (SABITA), 218502 Istanbul Medipol University, 34810 Istanbul, Turkey; § CSL Vifor Ltd., Redwood City, California 94063, United States; ∥ 159533 CSL Vifor Ltd., Flughofstrasse 61, 8152 Opfikon, Switzerland; ⊥ Center for Medical Research, CF Bioimaging, Medical University of Graz, Neue Stiftingtalstraße 6, 8010 Graz, Austria; # BioTechMed Graz, Mozartgasse 12/2, 8010 Graz, Austria

**Keywords:** iron reporter, iron carbohydrate nanoparticles, fluorescence microscopy, labile iron pool, Hr domain, iron-dependent degron

## Abstract

Here, we developed IronFist, a genetically encoded fluorescent reporter that enables dynamic tracking of labile ferrous ions (Fe^2+^) in live cells. IronFist is a bicistronic system combining the iron-sensitive hemerythrin-like (Hr) domain from the F-box and leucine-rich repeat protein 5 (FBXL5), fused to the bright fluorescent protein (FP) mNeonGreen, alongside mCherry as a reference FP signal. When labile iron levels are low, Hr-mNeonGreen undergoes ubiquitination and degradation, leading to a low green-to-red fluorescence ratio. Conversely, as cytosolic Fe^2+^ levels rise and Fe^2+^ ions bind to Hr, the green fluorescence is stabilized, increasing the IronFist ratio signal. Using IronFist for end point measurements, we observed that most cells maintain low basal labile iron levels. However, upon treatment with iron­(II) sulfate or iron carbohydrate nanoparticles, we detected significant elevations in the cellular labile iron pool (LIP). Cells responded faster and more strongly to iron­(II) sulfate, whereas responses to iron carbohydrate nanoparticles were slower and weaker. Time-lapse imaging further revealed substantial cell-to-cell heterogeneity in iron handling. We conclude that IronFist fills a critical methodological gap in assessing cellular iron homeostasis and related pathologies by enabling high-content tracking of iron dynamics at the single-cell level.

## Graphical abstract

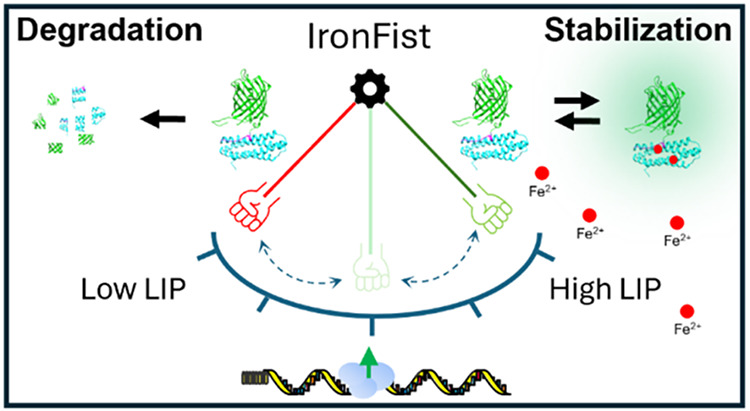

The ferrous ion (Fe^2+^) is a highly relevant trace element in biology, playing pivotal roles in numerous physiological processes and contributing to health and disease.^1,2^ Its great importance stems from its redox potential, which drives a wide range of biochemical processes, including—but not limited to—electron transport chains, oxygen transport, DNA repair, and cellular detoxification.^3–7^ Because of its thermodynamically favorable oxidation chemistry,^4^ Fe^2+^ can rapidly and nonenzymatically oxidize to ferric ions^8,9^ (Fe^3+^). This well-known Fenton reaction^3,7^ produces highly reactive hydroxyl radicals (OH^•^),^8^ which then can induce the peroxidation of polyunsaturated fatty acids in cells, ultimately leading to iron-mediated apoptosis, known as ferroptosis.^7–9^ Therefore, monitoring labile ferrous ions at the single-cell level can help assess the cellular iron status and predict cellular fate. However, the available chemical and genetically encoded iron indicators show limited sensitivity and no reversibility^10,11^ or can only indirectly gauge cellular labile Fe^2+^ content.^12^ To address the methodological gap in iron tracking in live cells, we built a next-generation genetically encoded fluorescent iron reporter system in this study.

Living systems employ sophisticated mechanisms to keep iron levels at a sharp balance^4,7,13,14^ and counteract the potential toxicity of bioavailable Fe^2+^. To achieve this balance in mammalian cells, iron-responsive elements (IREs) and iron regulatory proteins (IRPs) post-transcriptionally and reciprocally regulate the expression of the transferrin receptor (TfR), the primary iron uptake route in most cells,^15,16^ and ferritins, the key proteins involved in cellular iron storage.^17^ In this regulation, the expression of Aconitase2, one of the key IRPs, is controlled via its interaction with F-box and leucine-rich repeat protein 5 (FBXL5). Notably, the cellular abundance of FBXL5 is directly dependent on the free cytosolic Fe^2+^ concentration. While FBXL5 is stabilized under high cytosolic Fe^2+^, it gets targeted to proteasomal degradation under low cytosolic Fe^2+^. Thus, FBXL5, which directly binds free cellular Fe^2+^, has been recognized as an important physiological sensor of the labile iron pool (LIP) in mammalian cells.^4,18^

In this study, we harness these insights into the biological role and structure–function relationship of Fe^2+^-FBXL5 to bioengineer a bicistronic fluorescent iron reporter system capable of dynamically tracking labile Fe^2+^ in individual cells by fluorescence imaging.

## Results and Discussion

### Design and Functional Principle of IronFist: Revealing Low LIP in Most HeLa and EA.hy926 Cells

We developed a genetically encoded ratiometric green-red fluorescent iron reporter system named IronFist, which stands for Iron Fluorescent Indicator based on Stability and Translation (Figure 1A). To design IronFist based on FBXL5, we first examined the sequence and structure of its Hr domain, which contains the amino acid residues involved in di-iron coordination,^4^ and the iron-dependent degron sequence^4,19^ (Figure S1A and B). We hypothesized that the Hr domain of FXBL5, spanning amino acids 1 to 160, is thus sufficient to target a C-terminally fused protein of interest (e.g., an FP variant) for ubiquitination and proteasomal degradation under low cytosolic Fe^2+^ levels (Figure 1A). By using only the Hr domain, we avoided overexpression of full-length FBXL5 containing F-box domain and leucine-rich repeat domains (LRR1-LRR6),^3^ which could have unwanted effects on cell activities related to Aconitase2 ^3^ and other proteins.^20^ Moreover, to minimize the risk of overloading the proteasomal degradation machinery at low cytosolic Fe^2+^ levels, we employed the moderate human phosphoglycerate kinase (hPGK) promoter for expression (Figure 1A). The sensing component of IronFist consists of the Hr domain fused to mNeonGreen, a fast maturing (∼10 min) bright green FP variant,^21^ while the reference component is comprised of mCherry with comparable maturation kinetic (∼15 min), a red FP variant is expressed in an iron-independent manner via a ribosomal skipping sequence,^22,23^ allowing both the identification of positively transfected cells and ratiometric detection of the LIP (Figure 1A).

**Figure 1:**
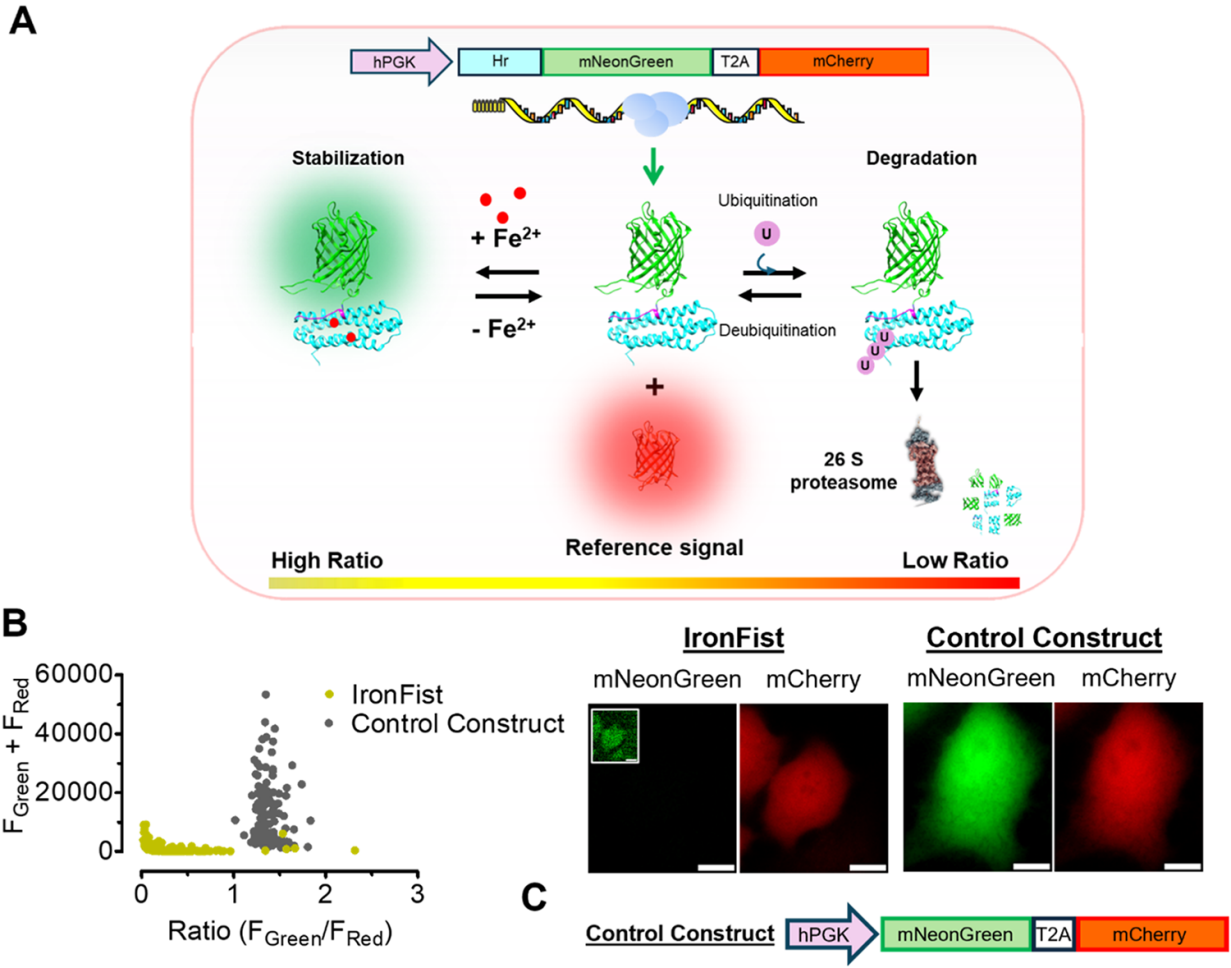
IronFist Design and Functionality for the ratiometric detection of LIP in living cells. (A) Cartoon representation of the reporter components and its mechanism of action. Using the T2A strategy two different fluorescent components are proportionally expressed, whereby the bright GFP variant mNeonGreen is fused to the Hr domain of FBXL5 and thus degraded via ubiquitination and proteasome machinery when labile iron is low, while mCherry, serves as a reference signal and is stable independently of the LIP. (B) Hr-mNeonGreen fusion protein efficiently degraded by HeLa cells in nontreated conditions. Left panel: scatter plots of *F*_Green_/*F*_Red_ ratio (*x* axis), versus expression (*F*_Green_ + *F*_Red_) HeLa cells expressing IronFist (dark yellow) and control construct (Control Construct, dark gray) in untreated conditions. Right panel: Representative images of analysis represented on the left panel. Data collected in two independent experiment, Control construct = 5 wells/164 cells, IronFist = 6 wells/68 cells. Expression of mNeonGreen in IronFist-expressing cells shown in smaller brightness contrast setting in inset image. Scale bars are 10 μm. (C) Cartoon representation of the iron insensitive control construct lacking Hr domain.

Upon an increase in cytoplasmic Fe^2+^ levels, ferrous ions bind to the Hr domain, stabilizing the Hr-mNeonGreen fusion protein by preventing its degradation. This results in a rise in the green-to-red ratio (Figure 1A), indicating elevated cytoplasmic LIP. On the other hand, under low cytoplasmic Fe^2+^ levels, the Hr-mNeonGreen fusion protein is degraded, reducing the green-to-red ratio. These changes in the ratio reflect the ability of IronFist to dynamically respond to fluctuations in cytosolic ferrous ions (Figure 1A), even though the reporter itself is not reversible in the classical sense, as it requires ongoing synthesis and is degraded under low-iron conditions. First, we transiently expressed IronFist in two cell lines, cancerous HeLa S3 cells and immortalized endothelial EA.hy926 cells, and used fluorescence microscopy to image the green and red fluorescence intensities of individual cells. Interestingly, many cells of both cell lines showed minimal visible mNeonGreen fluorescence, while the mCherry fluorescence was conspicuous (Figure 1B), suggesting that most individual cells exhibit a low LIP or the LIP under these conditions cannot be detected by IronFist. However, some HeLa cells showed higher green-to-red ratios (Figure 1B). To evaluate whether the fluorescence ratios are independent of IronFist expression levels under basal conditions, we plotted the sum of green and red fluorescence intensities for individual HeLa (Figure 1B, left panel) and EA.hy926 (Figure S2, left panel) cells against the corresponding green-to-red ratios. This analysis showed no correlation between ratio values and total expression levels (Figure 1B and Figure S2, left panel). A key advantage of IronFist appears to be its ability to detect low labile iron. Given the transient nature^24^ and toxicity of LIP, many cells are expected to maintain low levels of free Fe^2+^ under basal conditions, making it difficult to monitor LIP dynamics without a sensitive and specific reporter system like IronFist. To verify that the low green-to-red ratio of most cells indeed reflects low labile Fe^2+^, we compared the fluorescence ratios of IronFist-expressing cells with those expressing a control construct lacking the Hr domain (Figure 1B), rendering it insensitive to Fe^2+^. The Fe^2+^-insensitive control construct is made of the same hPGK promoter, ribosomal skipping sequence, and FP variants mNeonGreen and mCherry as in the IronFist (Figure 1C). In HeLa S3 or EA.hy926 cells expressing the control construct, we observed a green-to-red ratio around 1.4 with lower cell-to-cell variations due to a consistent mNeonGreen and mCherry expression (Figure 1B and Figure S2). These findings with IronFist and its respective control construct suggest that in cells expressing the IronFist, the efficient degradation of Hr-mNeonGreen fusion is enabled exclusively by the Hr domain.

To further test this assumption, we inhibited proteasomal degradation in IronFist-expressing cells using MG132, a known inhibitor of the proteasomal machinery.^25–27^ Consistent with our expectations, IronFist-expressing cells treated with MG132 exhibited elevated mNeonGreen fluorescence and green-to-red ratio signals (Figure S3). The mCherry fluorescence was not affected by MG132 (Figure S3), indicating that this FP variant is not targeted for MG132-sensitive proteasomal degradation. Overall, these data confirm the working mechanism of IronFist, which relies on proteasomal degradation (Figure 1A). Moreover, these data show that in HeLa and EA.hy926 cells, low mNeonGreen fluorescence results from efficient, continuous MG132-sensitive degradation of the Hr-mNeonGreen fusion construct, suggesting low labile cytosolic Fe^2+^ levels in most cells under these *in vitro* cell culture conditions.

### IronFist Detects Cellular Iron Uptake from Different Iron Sources

To assess whether IronFist can detect changes in the cellular iron status of a whole cell population, we generated a transgenic HeLa cell line stably expressing either IronFist or the control construct (Figure 1A, and C) and analyzed them by fluorescence-activated cell sorting (FACS) (Figure 2A). The experimental workflow, including lentivirus production, stable cell line generation, and subsequent flow cytometry analysis, is illustrated in Supplementary Figure S4. The FACS analysis of cells under basal conditions (untreated HeLa cells) shows that HeLa cells exhibit a much lower green-to-red (∼0.08) ratio signal with IronFist compared to the control construct (∼0.66) (Figure 2A, right panels), indicating a low average LIP in the HeLa cell population. Next, we treated the cells with Fe­(II) sulfate for 2 h and compared them with untreated cells. FACS analysis of cells stably expressing IronFist showed a significant increase, by nearly 2.5 fold, in mNeonGreen fluorescence in response to Fe­(II) sulfate treatment (Figure 2A, upper left panel). This shift in green fluorescence intensity was absent in the cell population stably expressing the control construct upon Fe­(II) sulfate addition (Figure 2A lower left panel). The mCherry fluorescence remained unchanged in both treated and untreated cells, regardless of whether they expressed IronFist or the control construct. Ratiometric FACS analysis thus showed that cell treatment with the Fe­(II) salt significantly increased the green-to-red ratio of the cell population stably expressing IronFist (Figure 2A upper right panel), but not that expressing the control construct (Figure 2A lower right panel). The increase in the IronFist green-to-red ratio (Figure 2A upper right panel), thus demonstrates that the tool effectively detects elevated levels of intracellular Fe^2+^ following Fe­(II) sulfate treatment in the HeLa cell population. Under these conditions, HeLa cells likely take up extracellular Fe^2+^ via the divalent metal transporter 1 (DMT1),^28,29^ spanning the plasma membrane, consequently leading to the stabilization of the cytosolic Hr-mNeonGreen construct and a corresponding rise in the IronFist green-to-red ratio signal, reporting an elevation of the cellular LIP.

**Figure 2:**
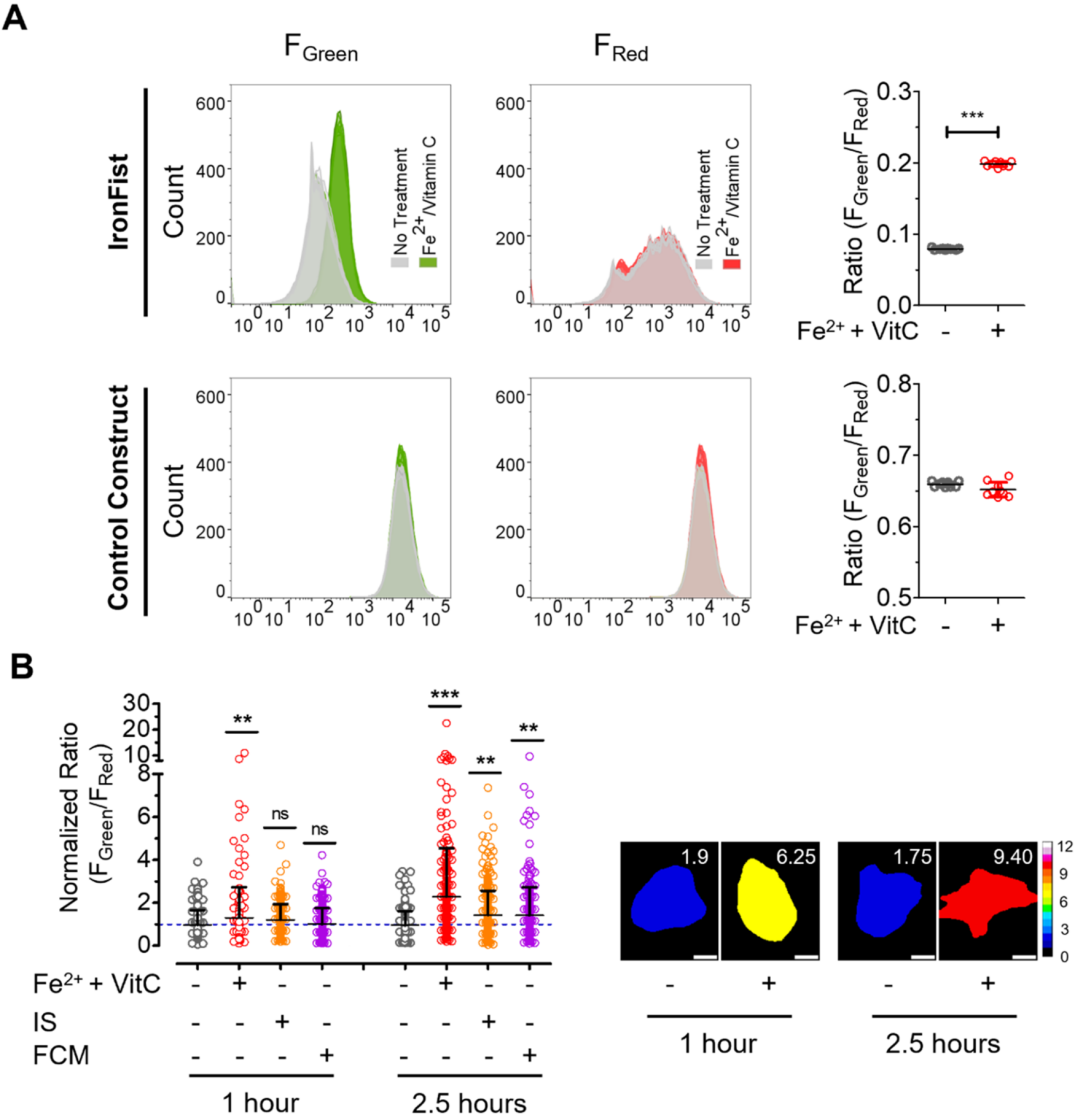
IronFist reports time-dependent elevations in LIP using FACS and single-cell microscopy. (A) FACS analysis of HeLa cells stably expressing IronFist (upper panels) and control construct (lower panels) with or without 150 μM FeSO_4_ and 250 μM Vitamin C treatment. Left to right: intensity distribution of the cells in green channel, red channel, and *F*_Green_/*F*_Red_ ratio plots. (B) IronFist reports LIP fluxes at end point by microscopy at single cell level. Left panel: Normalized *F*_Green_/*F*_Red_ ratio plots of HeLa cells transiently expressing IronFist treated with either 150 μM FeSO_4_ and 250 μM Vitamin C (Vit C; red), 500 μg iron/mL delivered as IS (Iron Sucrose; orange), or 500 μg iron/mL delivered as FCM (Ferric Carboxy Maltose; purple) for 1 and 2.5 h. Data were collected in three independent experiments. *n* of 1 h NT = 9 wells/240 cells, *n* of 1 h Fe+Vit C = 8 wells/185 cells, *n* of 1 h IS = 7 wells/179 cells, *n* of 1 h FCM = 9 wells/269 cells. *n* of 2.5 h NT = 9 wells/240 cells, *n* of 2.5 h Fe+Vit C = 9 wells/248 cells, *n* of 2.5 h IS = 9 wells/313 cells, *n* of 2.5 h FCM = 9 wells/256 statistical analysis performed for each time point separately with one-way ANOVA followed by Tukey′s multiple comparison ns: nonsignificant, **P < 0.001, ***P < 0.001, statistically significant. Right panel: representative ratio images of untreated cells and FeSO_4_+Vitamin C treated cells for both time points.

To assess cell-to-cell heterogeneity in response to extracellular Fe^2+^, we imaged IronFist in single HeLa cells using high-resolution fluorescence microscopy. Cells transiently expressing IronFist were treated for either 1 or 2.5 h with Fe­(II) sulfate. These end point measurements revealed substantial heterogeneity: while some cells showed no increase in the IronFist green-to-red ratio compared to untreated controls, others exhibited a pronounced increase that significantly exceeded the average ratio of control cells. This effect was detectable after 1 h and became even more pronounced after 2.5 h of Fe­(II) sulfate treatment (Figure 2B). Mechanistically, the addition of extracellular Fe^2+^ is expected to increase the intracellular LIP rapidly to its maximum. However, we observed a further increase in the IronFist ratio signal over time (Figure 2B). IronFist relies on the transcription and translation rates of its genetically encoded components, which might explain the delayed response in this experiment. To assess whether the observed response was cell-type specific, HEK 293 cells were treated with the same concentration of Fe­(II) sulfate for 2.5 h. This treatment resulted in a pronounced increase in the IronFist *F*_Green_/*F*_Red_ ratio in a substantial number of HEK 293 cells (Figure S5), similar to the response observed in HeLa cells (Figure 2B).

To prevent oxidation of Fe^2+^ to Fe^3+^ in an aqueous solution, Fe­(II) sulfate is supplemented with vitamin C throughout the study. To ascertain whether vitamin C alone influences the IronFist ratio, cells were treated with vitamin C without iron supplementation. Vitamin C alone did not alter the *F*_Green_/*F*_Red_ ratio at 2.5 h (Figure S6), suggesting that the observed ratio changes were specific to iron exposure in the Fe­(II) sulfate.

Based on the design of IronFist using a ribosomal skipping sequence under the same promoter, both IronFist components have the same rates of transcription and translation in cells transiently transfected. However, the transcription and translation rates might increase and then slow down over time after transfection. To assess how this might affect IronFist performance, we measured the green and red fluorescence intensities of single HeLa cells 24 and 42 h post-transfection, under no or Fe­(II) sulfate treatment conditions. In untreated cells, mNeonGreen fluorescence remained at the same low levels at both time points, while iron treatment led to a similar increase in fluorescence at 24 and 42 h (Figure S7A left panel). This indicates that Hr-mNeonGreen is efficiently degraded under basal conditions at 24 and 42 h post-transfection and becomes stabilized when intracellular Fe^2+^ levels increase upon iron treatment at both time points. However, mCherry fluorescence steadily increased from 24 to 42 h in both untreated and treated cells (Figure S7A middle panel), demonstrating its stability and continuous accumulation over time. Since the mCherry signal serves as a reference for expression levels, this increase leads to a lower green-to-red ratio at the later time point. Thus, the lower ratio at 42 h is not indicative of reduced LIP but rather reflects higher mCherry expression relative to Hr-mNeonGreen. This needs careful consideration for correct data interpretations.

Importantly, the green-to-red ratio increased upon iron treatment at both time points, demonstrating that the ratiometric IronFist reliably detects changes in intracellular Fe^2+^ over a broad time window (Figure S7A right panel). This assumption is further supported by our finding that the expression levels did not correlate with the ratio values at either time point after transfection (Figure S7B).

To investigate whether IronFist can detect time-dependent changes in LIP accumulation, despite a potential delay of the reporter system readout, we treated HeLa cells with iron carbohydrate nanoparticles: iron sucrose (IS) and ferric carboxy maltose (FCM). These clinically available intravenous (IV) iron carbohydrate nanoparticles are composed of polynuclear ferric oxyhydroxide cores bound to a carbohydrate ligand.^30,31^ Uptake likely occurs through endocytosis, followed by lysosomal biodegradation, where Fe^3+^ is released from the nanoparticles and Fe^3+^ is reduced to Fe^2+^. The lysosomal Fe^2+^ is then released into the cytosol via DMT1 ^28^ or other ion channels, such as TRPML1.^32^ Due to these distinct mechanisms of the uptake and biodegradation of iron carbohydrate nanoparticles, we expected a further delayed and weaker LIP increase compared to Fe­(II) sulfate. Indeed, IronFist detected a significant LIP rise only after 2.5 h of treatment with IS and FCM, which was also less pronounced than Fe­(II) sulfate. These experiments demonstrate that IronFist can resolve kinetic differences in LIP accumulation induced by cell treatments with distinct extracellular iron sources. To investigate whether in addition to the iron carbohydrate nanoparticles, ferric ammonium citrate (FAC), a commonly used Fe­(III) salt for supplying cells with bioavailable iron,^33–35^ can increase the labile iron pool, HeLa cells expressing IronFist were treated with either Fe­(II) sulfate plus vitamin C (150 and 250 μM, respectively) or an equivalent amount of iron delivered as FAC for 1 or 2.5 h (Figure S8). Both treatments induced a comparable and significant increase in the IronFist *F*_Green_/*F*_Red_ ratio (Figure S8). Given that, based on the known crystal structure,^19^ the Hr domain preferentially binds Fe­(II), these results rather suggest that FAC effectively supplies bioavailable iron and increases LIP, likely through cellular uptake of Fe­(III) followed by intracellular reduction to Fe­(II).

To validate that the IronFist signal increase upon Fe­(II) treatment results from specific iron binding rather than general effects on protein degradation, we engineered an iron-binding–deficient mutant (IronFist^MUT.^) by replacing all residues involved in coordinating the di-iron center, except histidine 80 in the degron region, with alanine (Figure S9A). To confirm that the mutations did not alter folding, we predicted the mutant’s 3D structure using AlphaFold2 and superimposed it with the wild-type Hr domain, revealing no structural deviations (Figure S9B). Functionally, Fe­(II) treatment did not increase the ratio in HeLa cells expressing the mutant, confirming its inability to bind iron. However, MG132 treatment significantly increased the ratio, indicating ongoing proteasomal degradation. These results confirm that the signal increase observed in wild-type IronFist requires functional iron binding (Figure S9C).

### Time-Lapse Imaging of IronFist Reveals Cell-to-Cell Variability in Iron Handling

To explore iron dynamics at the single-cell level, we performed long-term live-cell imaging of HeLa cells transiently expressing IronFist (Figure 3 and Figure S10). These experiments showed that ∼10% of cells exhibited no detectable response, probably because of impaired iron uptake or elevated storage and export of Fe­(II) ions under these conditions (Figure 3B). However, ∼30% of HeLa cells showed weak but significant elevations of the IronFist ratio signal, and ∼60% displayed a strong and sustained increase. Notably, 7 cells out of 34 strongly responding cells showed a transient signal (Figure 3A), indicating that under these conditions of high extracellular Fe­(II) some cells actively regulate elevated LIP, by increasing storage or export of Fe­(II) ions (Figure 3B).

**Figure 3:**
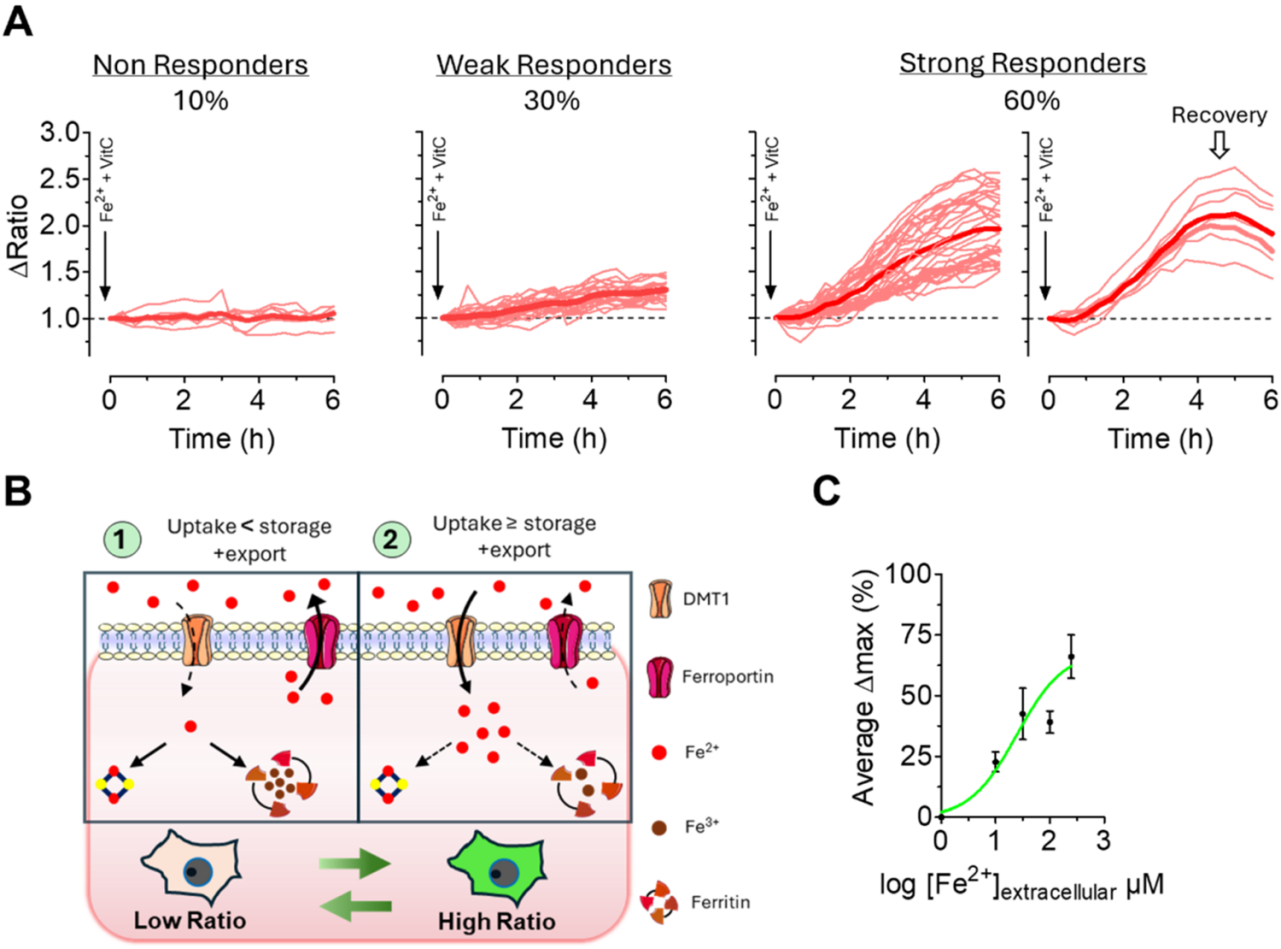
HeLa cells heterogeneous response to extracellular Fe­(II) sulfate and Vitamin C treatment, demonstrating variability in LIP dynamics. (A) Fe­(II) sulfate (250 μM) and Vitamin C (250 μM) treatment; 8 wells/69 cells: Left to right; nonresponders: Δ < 0.15, Cells maintain a stable ratio over the entire period, indicating no significant LIP increase (7 cells). Weak Responders; 0.15 ≤ Δ < 0.50, (21 cells). Strong Responders; Δ ≥ 0.50, Cells showing continuous increase in the ratio throughout the imaging, (34 cells). Strong Responders with recovery phase: Cells show an initial increase in the ratio, followed by a return phase (7 cells). Average curves are shown as thicker red lines. (B) Cartoon representation of possible scenarios and molecular mechanisms of observed differences in response to extracellular iron treatment. (C) Concentration–response of IronFist in HeLa cells to extracellular (10, 30, 100, 250 μM) Fe­(II) sulfate with equimolar Vitamin C Average Δmax ratios (% of a normalized ratio of 2 = 100%) were plotted against Fe­(II) sulfate concentrations. Data are average ± SEM (*n* = 3 wells/50 cells for 10 μM; 3/57 for 30 μM; 3/63 for 100 μM; 8/69 for 250 μM).

Next, we treated HeLa cells expressing IronFist with different extracellular Fe­(II) concentrations (Figure 3C and Figure S10). Even at 10 μM, we observed 10% of strong responders, and the proportion of responding cells and the onset kinetics increased with higher concentrations (Figure 3 and Figure S10). While 10 μM Fe­(II) sulfate might still reflect unphysiological conditions, these findings support the idea that IronFist can resolve subtle and rapid changes in iron uptake and handling at the single-cell level. However, we do not yet explicitly know if IronFist responds to very low physiological fluctuations of LIP. Nevertheless, we observed distinct response patterns, which highlight the substantial heterogeneity in iron handling among individual cells. However, additional experiments are needed to explore further the molecular mechanisms underlying these responses and validate the predictive power of IronFist in various disease models and therapeutic contexts.

## Conclusion

In this study, we developed IronFist, a genetically encoded fluorescent reporter that harnesses the physiological function of FBXL5 in the regulation of cellular iron homeostasis. By leveraging this central pathway in counteracting iron toxicity, IronFist provides a dynamic and reversible response to changes in LIP, translating it into an optical signal. IronFist thus enables the visualization of shifts in iron status at the single-cell level before iron toxicity occurs. Notably, IronFist is not a biosensor in the traditional sense, as Fe^2+^ binding neither instantly changes the fluorescence intensity nor the spectral properties of the FP but prevents its degradation. Based on the IronFist principle, this reporter system requires biochemically active cells with ongoing transcription, translation, and protein degradation processes, which limits its calibration. However, we believe that IronFist represents an important sensing technology that holds significant potential for assessing the ferroptotic risks associated with drugs that interfere with iron homeostasis or increase LIP. Our findings demonstrate that cells can compensate for fluctuating free iron in the cytoplasm, emphasizing the dynamic nature of cellular iron regulation. This ability to monitor and identify compensatory mechanisms opens new avenues for understanding the fine balance between iron homeostasis and toxicity. Future studies can use IronFist to probe these mechanisms in more detail and explore potential therapeutic interventions that modulate cellular iron levels in medication and disease contexts.

## Experimental Section

Detailed materials and methods are described in Supporting Information.

### Buffers and Solutions

Cell culture materials were obtained from Grainer Bio-One (Kremsmünster, Austria). EH loading buffer prepared in-house.

### Compounds

IS, (iron sucrose nanoparticle) and FCM (Ferric carboxy maltose nanoparticle) were kind gifts from CSL Vifor, St. Gallen, Switzerland. The rest of the compounds used in this study are described in Supporting Information file.

### Molecular Cloning and Design of IronFist

Both IronFist (Addgene # 243006), IronFist^MUT.^ (Addgene # 243008), and control construct (Addgene # 243007) carrying vectors have been synthesized by Vector Builder (Germany).

### Lentivirus Production

For lentivirus generation, constructs were subcloned into a third-generation lentivirus shuttle vector pLenti-MP2 (Addgene #36097), and HEK293T cells were used for lentivirus generation as described previously.^36^

### Cell Culture and Transfection

HeLa S3, EA. hy926, and HEK 293 cells were cultured in complete medium and were routinely passaged every 2–3 days and kept in a humidified cell culture incubator (37 °C 5%CO_2_). For end point measurements, cells were seeded on 30 mm glass coverslips in 6-well plates. For time-lapse imaging, HeLa cells were seeded in Cellvis 12-well glass-bottom plates. One day after seeding, cells were transiently transfected with respective plasmids with Polyjet and imaged after 36–42 h.

### Stable Cell Line Generation

To generate stable cell lines, cells were initially seeded in a 6-well plate. at 50–60% confluency, transduction was performed using the appropriate lentivirus. Cells expressing the desired plasmids were then isolated using fluorescence-activated cell sorting (FACS).

### Iron Treatment and FACS Analysis

HeLa cells stably expressing IronFist or Construct constructs were treated with iron solution under identical conditions. Flow cytometry analysis was conducted using a BD FACSymphony A1 Cell Analyzer (BD Biosciences).

### End Point and Time-Lapse Fluorescent Microscopy Imaging

Transiently transfected HeLa S3 and EA.hy926 cells imaged with Nikon Eclipse Ti2 (Nikon, Austria) in EPI-Fluorescent mode. The microscope configuration is described in detail in the Supporting Information.

### Image Analysis

Images processed by Fiji software. For two channel intensity calculations, a macro script was used. Ratio images were generated by using each cell’s Roi and the ratio assigned by the math function of Fiji.

### Data Analysis

The data obtained from microscope images were transferred and analyzed in GraphPad Prism 5 Software (GraphPad Software, Inc., La Jolla, CA, USA).

## Supplementary Material



## Notes

Figure S1: The structure of the FBXL5 and its Hr domain; Figure S2: Hr-mNeonGreen fusion protein efficiently degraded by an EA.hy926 cells in nontreated conditions; Figure S3: Response of IronFist to Proteasome Inhibition with 10 μM MG132 for 6 h in HeLa Cells; Figure S4: Schematic Overview of Lentiviral Vector Design, Stable Cell Line Generation, and Flow Cytometry Analysis; Figure S5: IronFist responds to Fe­(II) sulfate + Vitamin C treatment in HEK 293 cells; Figure S6: IronFist is not affected by Vitamin C treatment; Figure S7: IronFist expression and ratiometric signal at 24- and 42 h post-transfection; Figure S8: IronFist response to Fe­(II) sulfate and Fe (III) ammonium citrate treatment in HeLa cells; Figure S9: Functionality of IronFist Fe^2+^ binding deficient mutant; Figure S10: Dose-dependent IronFist responses in HeLa cells treated with Fe­(II) sulfate and vitamin C (PDF)
