# Supplementary material for: Tracking Intracellular Labile Iron with a Genetically Encoded Fluorescent Reporter System Based on Protein Stability
Source: ACS Sens. 2025 Aug 1;10(8):5854–61. doi: 10.1021/acssensors.5c01165 (PMC12379171; doi:10.1021/acssensors.5c01165)
Supplement: Supplementary file 1 [file se5c01165_si_001.pdf]

Supplementary Information to

Tracking Intracellular Labile Iron with a Genetically Encoded Fluorescent Reporter System Based on Protein Stability

Ali Akyol[1], Şeyma Çimen[2], Benjamin Gottschalk[1], Yusuf C. Erdoğan[1], Anna Lischnig[1] Amy Barton Alston[3], Reinaldo Digigow[4], Beat Flühmann[4], Emrah Eroğlu[2,5], Wolfgang F. Graier[1,6], and Roland Malli,[5,6*]

[1]Gottfried Schatz Research Center, Molecular Biology and Biochemistry, Medical University of Graz, Neue Stiftingtalstraße 6, 8010 Graz, Austria
[2]Regenerative and Restorative Medicine Research Center (REMER), Research Institute for Health Sciences and Technologies (SABITA), Istanbul Medipol University, 34810 Istanbul, Turkey
[3]CSL Vifor Ltd., Redwood City, CA, 94063 USA
[4]CSL Vifor Ltd., Flughofstrasse 61, 8152 Opfikon, Switzerland
[5]Center for Medical Research, CF Bioimaging, Medical University of Graz, Neue Stiftingtalstraße 6, 8010 Graz, Austria
[6] BioTechMed Graz, Mozartgasse 12/2, 8010 Graz, Austria

*Lead contact and correspondence: roland.malli@medunigraz.at

Figure S1: The structure of the FBXL5 and its Hr domain.

Figure S2: Hr-mNeonGreen fusion protein efficiently degraded by EA. hy926 cells in non-treated conditions.

Figure S3: Response of IronFist to Proteasome Inhibition with 10 µM MG132 for 6 hours in HeLa Cells.

Figure S4: Schematic Overview of Lentiviral Vector Design, Stable Cell Line Generation, and Flow Cytometry Analysis.

Figure S5: IronFist responds to Fe (II) sulfate + Vitamin C treatment in HEK 293 cells.

Figure S6: IronFist is not affected by Vitamin C treatment.

Figure S7: IronFist expression and ratiometric signal at 24- and 42-hours post-transfection.

Figure S8: IronFist response to Fe (II) sulfate and Fe (III) ammonium citrate treatment in HeLa cells.

Figure S9: Functionality of IronFist Fe^{2+} binding deficient mutant.

Figure S10: Dose-dependent IronFist responses in HeLa cells treated with Fe (II) sulfate and vitamin C.

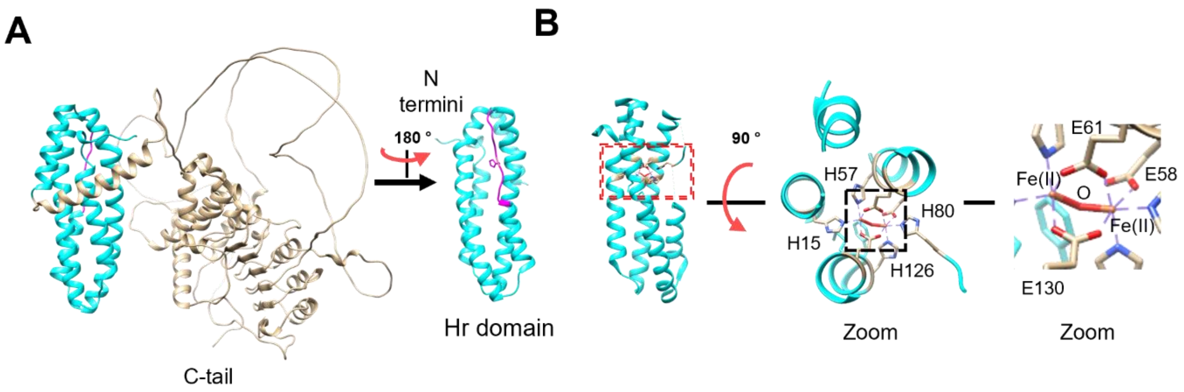

Figure S1 | The structure of the FBXL5 and its Hr domain. (**A**) Left panel: Alpha fold prediction of full length FBXL5 (taken from Uniprot: AF-Q9UKA1-F1.) Right panel: Hr domain of the protein (1-160 aa.), degron sequence (75-85 aa.) shown in magenta. (**B**) Left panel: Crystal structure of the Hr domain (taken from PDB PDB:3V5X). Middle and right panels zoom in on a di-iron binding pocket.

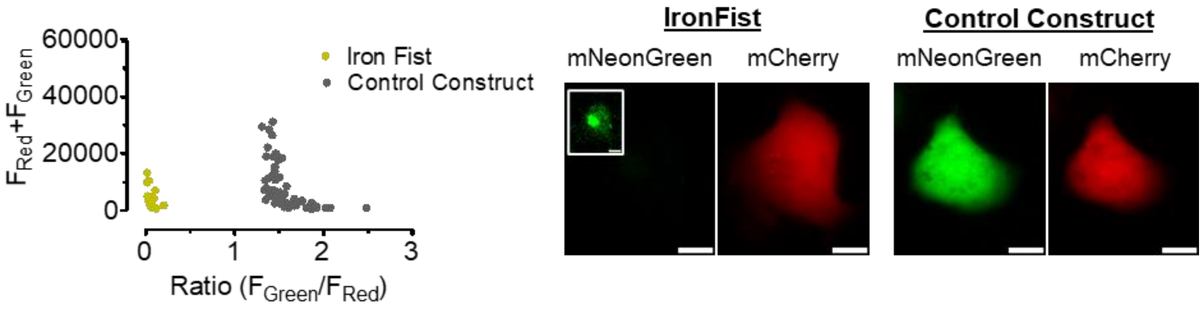

Figure S2 | Hr-mNeonGreen fusion protein efficiently degraded by and EA. hy cells in non-treated conditions. Left panel: F_{Green}/F_{Red} (x axis), versus expression ($F_{Green} + F_{Red}$) plots of the EA. hy926 cells expressing IronFist (dark yellow) and control construct (Control Construct, grey) **Right panel:** Representative images of analysis represented on the left panel. Data collected in two independent experiment, Control construct = 6 wells/ 74 cells, IronFist = 6 wells/25 cells. Expression of mNeonGreen in IronFist expressing cells shown in smaller brightness contrast setting in inset image. Scale bars are 10μm.

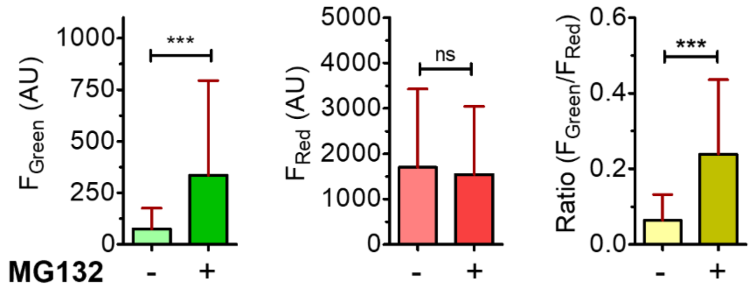

Figure S3 | Response of IronFist to Proteasome Inhibition with 10 μM MG132 for 6 hours in HeLa Cells. Left panel: mNeonGreen intensity plots, **middle panel:** mCherry intensity plots, **right panel:** F_{Green}/F_{Red} plots. Data collected in three independent experiments with triplicate n of control = 8 wells/324 cells, MG132 6h=9 wells/337 cells, DATA=Mean + SD, statistical analysis nonparametric t test. ***P<0.001 statistically significant ns: non-significant.

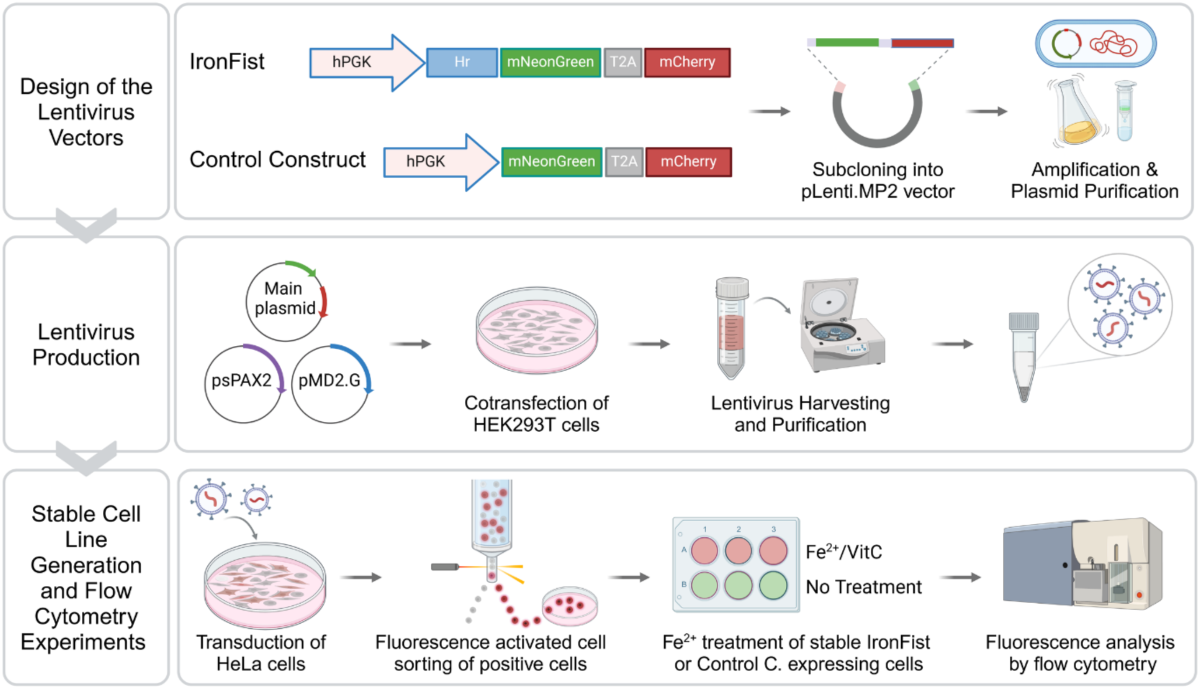

Figure S4 | Schematic Overview of Lentiviral Vector Design, Stable Cell Line Generation, and Flow Cytometry Analysis: Top panel: The workflow of subcloning of IronFist and control construct into the pLenti.MP2 vector. **Middle panel:** Lentiviral production process by co transfecting HEK293T cells with the main construct and packaging plasmids (psPAX2 and pMD2.G) and virus harvesting steps after 48- and 72-hours post-transfection. **Bottom panel:** HeLa S3 stable cell line generation. cells were transduced with the purified lentivirus, and fluorescence-activated cell sorting (FACS) was performed to isolate mCherry-positive cell populations. Stable cells expressing IronFist or the control construct were then treated with Fe^{2+}+ Vitamin C for 2 hours or left untreated before fluorescence analysis via flow cytometry.

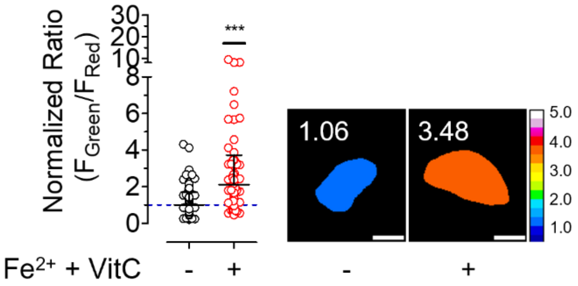

Figure S5 | IronFist responds to Fe (II) sulfate + Vitamin C treatment in HEK 293 cells. Left panel: Normalized Ratio plots (F_{Green}/F_{Red}) of HEK293 cells transiently expressing IronFist. n of NT= 3 wells/135 cells, n of Fe^{2+} + VitC = 3 wells/118 cells. **Right panel:** Representative pseudo coloured ratio images of analysis represented on the left panel. Statistical analysis nonparametric t test. ns: non-significant, ***P<0.001 statistically significant. Scale bars are 10 μm.

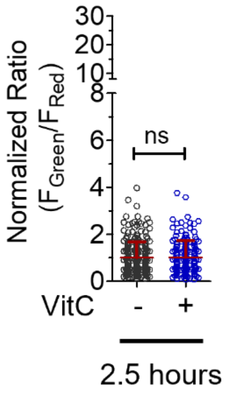

Figure S6 | IronFist is not affected by Vitamin C treatment. Normalized F_{Green}/F_{Red} ratio plots of HeLa cells expressing IronFist treated with 250 µM Vitamin C (blue) compared to untreated cells (dark grey). Data collected from three independent experiments with triplicates n of NT= 9 wells/244 cells, n of VitC= 7 wells/160 cells. DATA = Mean + SD, statistical analysis non-parametric t test, ns: non-significant.

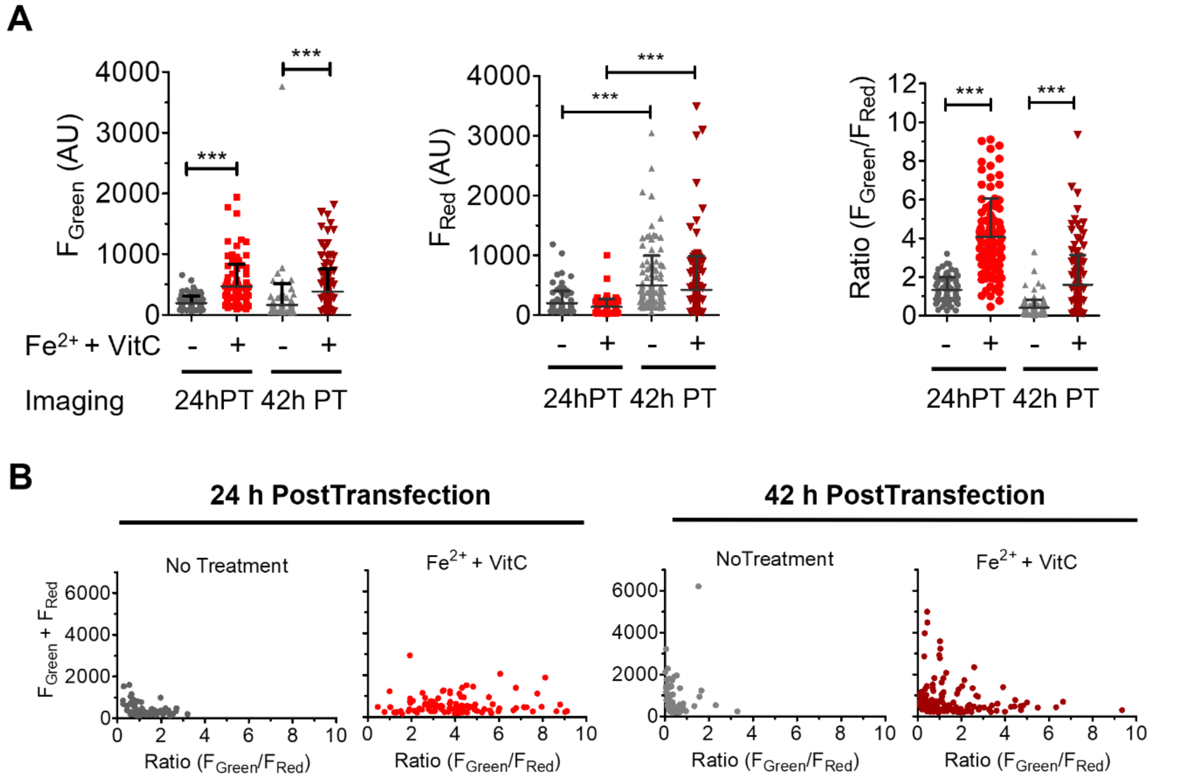

Figure S7 | IronFist expression and ratiometric signal at 24 and 42 hours post-transfection. (A) Both fluorescent channel intensities and F_{Green}/F_{Red} Ratio plots at 24 hours, and 42 hours post transfection (PT). **Left panel**: Distribution of mNeonGreen intensities of each cell is displayed: the first dark grey dots represent untreated cells at 24 hours post-transfection, followed by the second light red squares for treated cells at the same time point. The next two columns show data for 42 hours post-transfection, with grey for untreated cells and red for treated cells. **Middel panel:** Displays corresponding values for the mCherry reference protein. **The right panel:** F_{Green}/F_{Red} ratios for both time points are shown, comparing untreated (grey) and treated cells (red) to illustrate changes in iron sensitivity. (**B**) Scatter plots display the F_{Green} + F_{Red} sum (Y) plotted against the F_{Green}/F_{Red} values (X) of all the individual cells at both 24 (left) and 42 (right) hours, highlighting the absence of correlation between expression levels and ratio values. Data collected from two independent experiments with triplicates. n of 24h PT No treatment= 6 wells/80 cells, n of 24h PT Fe^{2+} + VitC= 6 wells/94 cells, n of 42h PT No treatment= 6 wells/125 cells, n of 42h PT Fe^{2+} + VitC= 6 wells/132 cells. DATA = Mean + SD, statistical analysis nonparametric t test ns: non-significant, ***P<0.001 statistically significant.

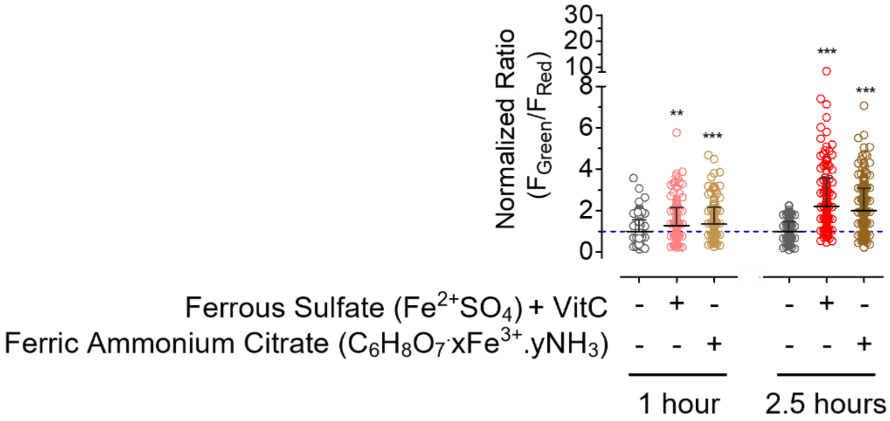

Figure S8 | IronFist response to Fe (II) sulfate and Fe (III) ammonium citrate treatment in HeLa cells. HeLa cells transiently expressing the IronFist were treated with either Fe (II) sulfate + vitamin C (150 and 250 µM, respectively) or with an equivalent amount of iron delivered as ferric ammonium citrate (FAC; $C_6H_8O_7.xFe^{3+}.yNH_3$) for 1 and 2.5 hours. n of No Treatment 1 h (light grey) = 4wells/200 cells, n of Fe (II) sulfate + vitamin C 1h (light red) = 4wells/177 cells, n of FAC 1h (light brown) = 4wells/175 cells, n of No Treatment 2.5 h (dark grey) = 4wells/221 cells, n of Fe (II) sulfate + vitamin C 2.5h (dark red) = 4wells/197 cells, n of FAC 2.5h (dark brown) = 4wells/241 cells. statistical analysis performed for each time point separately with 1way ANOVA followed by Tukey`s multiple comparison. ns = non-significant, **P<0.001, ***P<0.001, statistically significant.

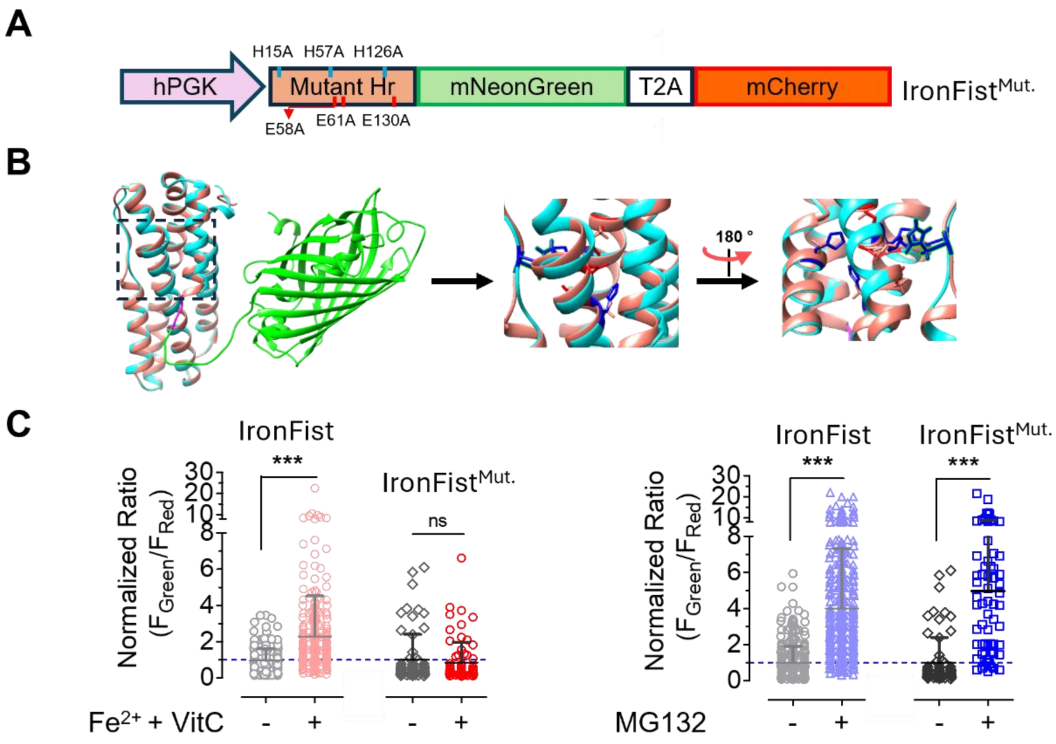

Figure S9 | Functionality of IronFist Fe^{2+} binding deficient mutant (A) Cartoon representation of IronFist$^{MUT.}$ with indicated mutations on the iron binding pocket. **(B)** Structure comparison of Wild type Hr domain and iron binding mutant. **Left panel:** super imposition of wild type Hr domain, cyan (taken from Uniprot: AF-Q9UKA1-F1.) and Alfa fold 2.0 predicted structure of iron binding mutant IronFist, orange. **Middle panel:** zoom in to the di-iron binding Center. **Right panel:** 180 ° rotation of middle panel on y-axis. **(C) Left panel:** Comparison of IronFist and IronFist mutant response to $FeSO_4$ (150 µM) + Vitamin C (250 µM), 2.5 hours. **Right panel:** Comparison of IronFist and IronFist mutant response to MG132 (10µM), 6 hours treatment. For mutant construct, data collected from two independent experiment. n of No treatment = 4wells/68 cells, n of 2,5h Fe^{2+}+VitC = 4wells/74 cells, n of MG132 (10µM) = 4wells/64 cells. Iron Fist data shown previous experiment and plotted here for comparison. Statistical analysis nonparametric t test ns = non-significant, ***$P<0.001$, statistically significant.

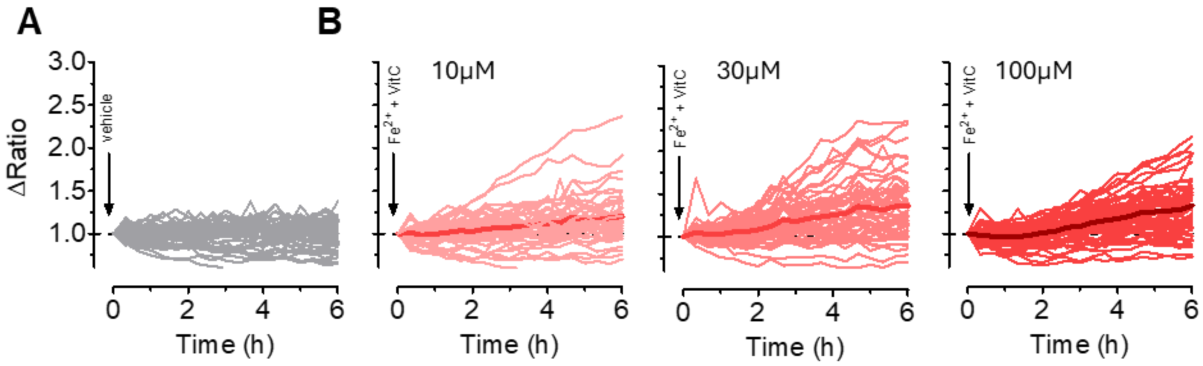

Figure S10 | Dose-dependent IronFist responses in HeLa cells treated with Fe (II) sulfate and vitamin C. HeLa cells transiently expressing the IronFist were treated with $FeSO_4$ at increasing concentrations (10, 30, 100 μM), each supplemented with an equimolar concentration of vitamin C, and imaged over six hours. Normalized ratio curves (normalized to t=0) reveal distinct labile iron pool (LIP) dynamics across single cells. Data were collected in triplicates. **(A) No Treatment:** n = 3 wells/50 cells. **(B) Left panel (10 μM):** n = 3wells/50 cells; non-responders, (52.0 %); weak responders, (38 %); strong responders, (10 %). **Middle panel (30 μM):** n = 3wells/57 cells; non-responders, (35.1 %); weak responders, (29.8 %); strong responders, (35.1 %). **Right panel (100 μM):** n = 3wells/63 cells; non-responders, (30.2 %); weak responders, (41.2 %); strong responders, (28.6 %). Average response curves for each concentration are overlaid as thick red lines. $\Delta < 0.15$ = non-responders; $0.15 \leq \Delta < 0.50$ = weak responders; $\Delta \geq 0.50$ = strong responders.

Materials and Methods

Buffers and Solutions: Cell culture materials were obtained from Grainer Bio-One (Kremsmünster, Austria). EH loading buffer prepared in house with following ingredients and concentrations (in mM); 2 $CaCl_2$, 135 NaCl, 1 $MgCl_2$, 5 KCl, 10 HEPES, 2.6 $NaHCO_3$, 0.44 KH_2PO_4, 0.34 Na_2HPO_4, 10 D-Glucose, 2 L-Glutamine and further supplemented with 1x amino acids, 1x vitamins (gibco REF:11130-036 and, gibco REF:11120-037, respectively. Then the pH of the solution was adjusted to 7.45 by NaOH.

Compounds: MG 132 purchased from MedChem Express (#HY-13259) and dissolved in DMSO at 10 mM concentration then aliquoted and kept at -20 °C for further use and used at 10 μM final concentration. IS, (iron sucrose nanoparticle) and FCM (ferric carboxy maltose nanoparticle) were kind gifts from CSL Vifor, St. Gallen, Switzerland. Ferrous sulfate heptahydrate was purchased from Sigma Aldrich #215422. Ferric ammonium citrate obtained from Sigma Aldrich #F5879. Ascorbic acid is obtained from Aponorm. $FeSO_4$ + Vitamin C mix was prepared freshly by dissolving both compounds in double distilled water or EH loading buffer.

Molecular Cloning and design of pseudo ratio metric IronFist: To generate a ratio metric tool we have designed a vector which has a fusion of Hr domain (NCBI Reference Sequence: NM_012161.4, nt:1-480) to a bright version of GFP (mNeonGreen) followed by a ribosomal skipping sequence, T2A, and mCherry fluorescent protein as a reference signal. As a non-iron dependent controls, we also designed a control vector that expresses both fluorescent proteins under the same hPGK (human phosphoglycerate kinase) promoter. Additionally, we have designed an iron binding mutant (IronFist$^{MUT.}$). All vectors have been synthesized by Vector Builder (Germany).

Lentivirus production

For lentivirus generation, constructs were subcloned into a 3rd-generation lentivirus shuttle vector pLenti-MP2 (Addgene #36097), and HEK293T cells were used for lentivirus generation[33]. Briefly, when cells reached 80–90% confluency, they were co-transfected with 3 μg psPAX2 (Addgene #12260), 3 μg pMD2.G (Addgene #12259), and 6 μg of the respective construct using the PolyJet (Signagen Laboratories, Rockville, MD, USA) transfection reagent. After 24 hours, the transfection medium was replaced with fresh DMEM. The virus-containing medium was collected at 48 and 72 hours post-transfection, filtered through a 0.45 μm low protein-binding filter (T.P.P., Switzerland), and concentrated using the sucrose cushion method. The purified viral particles were aliquoted and stored at –80 °C for subsequent stable cell line generation.

Cell Culture and Transfection: Hela S3, EA. hy926 and HEK 293 cells were cultured in Dulbecco's modified Eagle medium (DMEM D5523, Sigma Aldrich) supplemented with 10% FCS, 1% penicillin-streptomycin, 1.25mg/mL amphotericin B, and 25 mM HEPES (pH=7.45). Cells were routinely passaged every 2-3 days and kept in a humidified cell culture incubator (37°C 5%CO_2). Before the experiment, cells were seeded on 30mm glass coverslips (Co. KG, Lauda-Königshofen) in 6 well plates (Paul Marienfeld GmbH, Germany). For time-lapse imaging, HeLa cells were seeded on Cellvis 12 well glass bottom plates (P12-1.5H-N, IBL Baustoff+Labor GmbH, Wien, Austria). One day after seeding cells were transiently

transfected with respective plasmids with Polyjet according to the manufacturer's manual. Cells were imaged 36-42 hours after transfection in EH loading buffer supplemented with 1 % FCS.

Stable Cell Line Generation: To generate stable cell lines, cells were initially seeded in a 6-well plate. Once they reached 50-60% confluency, transduction was performed using the appropriate lentivirus in an antibiotic-free medium supplemented with 10% FBS and 10 µg/ml Polybrene for 48-72 hours. Cells expressing the desired plasmids were then isolated using fluorescence-activated cell sorting (FACS). Specifically, mCherry-expressing cells were sorted with a 561 nm laser (Filter: 593/40 nm) using a BD Influx Cell Sorter.

Iron Treatment and FACS Analysis: FACS analysis was conducted to assess the IronFist response to iron treatment at 2 hours time point. A HEPES-buffered solution was used for both iron treatment preparation and FACS analysis. This solution contained 2 mM $CaCl_2$, 5 mM KCl, 138 mM NaCl, 1 mM $MgCl_2$, 10 mM HEPES, and 10 mM D-glucose, with the pH adjusted to 7.42 using 1 M NaOH. Iron(II) supplementation was prepared by dissolving 150 µM $FeSO_4$ and 250 µM Vitamin C in the same buffer.
HeLa cells stably expressing IronFist or Control Construct were treated with iron solution under identical conditions. After treatment, cells were trypsinized, washed, and resuspended in the HEPES-buffered solution. For the negative control group, cells were incubated solely in the HEPES buffer without iron supplementation. Flow cytometry analysis was conducted using a BD FACSymphony A1 Cell Analyzer (BD Biosciences) with a 480 nm laser and a 530/30 nm bandpass filter for mNeoGreen and a 561 nm laser with a 586/15 nm bandpass filter for mCherry. Data were collected from a minimum of 10,000 viable cells per sample and analyzed using FlowJo 10.10.0 software (BD). Appropriate gating strategies were implemented to eliminate debris and doublets, ensuring accurate measurement of fluorescence signal variations across time points.

End Point Live Cell Imaging: Transiently transfected HeLa S3 and EA.hy926 cells imaged with Nikon Eclipse Ti2 (Nikon, Austria) in EPI-Fluorescent mode, equipped with CoolLED pE800 light source (CoolLED, UK, Andover), LED light source with 365, 400, 435, 470, 500, 550, 580, 635, and 740 nm (LEDs is introduced at the back focal plane), Apo 40X/1.15NA water immersion objective, two back-illuminated Kinetix Scientific CMOS cameras (TELEDYNE PHOTOMETRICS, USA, Tucson) mounted to the Nipkow based Crest optics X Light V3 with 70µm pinhole spinning disc system (Crestoptics, Italy, Rome) in EPI fluorescent mode by bypassing the spinning disc. The Nikon software (NIS-Elements AR 5.42.06 (Build 1821) LO 64bit, Nikon, Austria) was used for microscope control and image acquisition.

Image Analysis: Images were processed via Fiji software. For two-channel intensity calculations a macro script was used. First, the background is subtracted by using a rolling ball function. ROIs (regions of interest) were manually drawn around the cells, and respective fluorescence intensities were exported. Finally, the ratio of and sum of both channels (F_{Green}/F_{Red}, and F_{Green} + F_{Red}, if applicable) were calculated in Microsoft Excel. Ratio images were generated by using each cell's Roi and the ratio assigned by the math function of Fiji.

Data Analysis: The data obtained from the experiments were transferred and further analyzed in GraphPad Prism 5 Software (GraphPad Software, Inc., La Jolla, CA, USA)
